# Micro-Inclusion Engineering via Sc Incompatibility for Luminescence and Photoconversion Control in Ce^3+^-Doped Tb_3_Al_5−x_Sc_x_O_12_ Garnet

**DOI:** 10.3390/ma17112762

**Published:** 2024-06-05

**Authors:** Karol Bartosiewicz, Robert Tomala, Damian Szymański, Benedetta Albini, Justyna Zeler, Masao Yoshino, Takahiko Horiai, Paweł Socha, Shunsuke Kurosawa, Kei Kamada, Pietro Galinetto, Eugeniusz Zych, Akira Yoshikawa

**Affiliations:** 1 Faculty of Physics, Kazimierz Wielki University, Powstańców Wielkopolskich Street 2, 85-090 Bydgoszcz, Poland; 2 Institute of Low Temperature and Structure Research, Polish Academy of Sciences, 50-422 Wrocław, Poland; r.tomala@intibs.pl (R.T.); d.szymanski@intibs.pl (D.S.); 3 Department of Physics, University of Pavia, Via Bassi 6, 27100 Pavia, Italy; benedetta.albini@unipv.it (B.A.); pietro.galinetto@unipv.it (P.G.); eugeniusz.zych@uwr.edu.pl (E.Z.); 4 Faculty of Chemistry, University of Wrocław, Joliot-Curie Street 14 F, 50-383 Wrocław, Poland; justyna.zeler@uwr.edu.pl; 5 New Industry Creation Hatchery Center, Tohoku University, Sendai 9808577, Japan; masao.yoshino.a5@tohoku.ac.jp (M.Y.); takahiko.horiai.a5@tohoku.ac.jp (T.H.); shunsuke.kurosawa.a6@tohoku.ac.jp (S.K.); kei.kamada.c6@tohoku.ac.jp (K.K.); akira.yoshikawa.d8@tohoku.ac.jp (A.Y.); 6 Łukasiewicz Research Network—Institute of Microelectronics and Photonics, Aleja Lotników 32/46, 02-668 Warsaw, Poland; psocha.sci@gmail.com; 7 Institute for Materials Research, Tohoku University, 2-1-1 Katahira, Sendai 9808577, Japan; 8 Institute of Laser Engineering, Osaka University, 2-6 Yamadaoka, Osaka 5650871, Japan; 9 C&A Corporation, 1-16-23 Ichibancho, Sendai 9800811, Japan

**Keywords:** energy transfer, Ce^3+^ luminescence, Tb^3+^ luminescence, photoconversion, white LED, Raman spectroscopy, thermoluminescence, single crystal, garnet, perovskite

## Abstract

Aluminum garnets display exceptional adaptability in incorporating mismatching elements, thereby facilitating the synthesis of novel materials with tailored properties. This study explored Ce^3+^-doped Tb_3_Al_5−x_Sc_x_O_12_ crystals (where x ranges from 0.5 to 3.0), revealing a novel approach to control luminescence and photoconversion through atomic size mismatch engineering. Raman spectroscopy confirmed the coexistence of garnet and perovskite phases, with Sc substitution significantly influencing the garnet lattice and induced A_1g_ mode softening up to Sc concentration x = 2.0. The Sc atoms controlled sub-eutectic inclusion formation, creating efficient light scattering centers and unveiling a compositional threshold for octahedral site saturation. This modulation enabled the control of energy transfer dynamics between Ce^3+^ and Tb^3+^ ions, enhancing luminescence and mitigating quenching. The Sc admixing process regulated luminous efficacy (LE), color rendering index (CRI), and correlated color temperature (CCT), with adjustments in CRI from 68 to 84 and CCT from 3545 K to 12,958 K. The Ce^3+^-doped Tb_3_Al_5−x_Sc_x_O_12_ crystal (where x = 2.0) achieved the highest LE of 114.6 lm/W and emitted light at a CCT of 4942 K, similar to daylight white. This approach enables the design and development of functional materials with tailored optical properties applicable to lighting technology, persistent phosphors, scintillators, and storage phosphors.

## 1. Introduction

Aluminum garnets belong to the group of multifunctional materials due to their exceptional structural stability and compositional flexibility [1,2,3,4,5,6,7,8,9,10,11]. This group of compounds exhibits a high degree of tolerance for doping ability and readily accepts a wide range of rare earth and/or transition metals [4,8,9,12]. Aluminum garnets also exhibit a highly ordered, three-dimensional cubic lattice structure characterized by exceptional thermal stability, exhibiting no phase transitions across an extensive temperature range [13]. The absence of phase transitions in aluminum garnets, unlike in many materials (e.g., perovskites) that undergo structural changes at different temperatures, guarantees consistent material properties across a wide temperature range. This translates to a significant simplification of the crystal growth process from the melt and transparent ceramics sintering process, as the lattice structure does not change the phase during solidification, leading to crack-free material with high mechanical strength [2,3,8,9]. Therefore, aluminum garnets are important materials in diverse fields, including laser materials [14], scintillators [2,15], persistent luminescence phosphors [9,10], and photoconverters [1,8,11].

The exceptional combination of strong blue absorption and broad yellow emission of Ce^3^⁺ ions in the Y_3_Al_5_O_12_ (YAG) matrix significantly enhances their suitability as a phosphor for blue-LED-based white LEDs. High optical absorption (>95%) and quantum efficiency (>83% under excitation in the 440–470 nm spectral range) further enhance its advantageous features [16]. However, Ce^3+^-doped Y_3_Al_5_O_12_-based white LEDs exhibit limitations in achieving the desired color rendering. This type of optical material is characterized by a low color rendering index (CRI) and a high correlated color temperature (CCT > 5000 K), making it challenging to generate a warm white light. The above spectroscopic parameters for the Ce^3+^-doped Y_3_Al_5_O_12_ result from the low-intensity emission recorded in the red spectral range [17]. One frequent approach to tuning the emission color of Ce^3+^-doped Y_3_Al_5_O_12_ involves the substitution of yttrium with other rare-earth elements within the Y_3_Al_5_O_12_ crystal lattice. This tune involves either the partial or complete replacement of Y^3+^ sites with larger cations, such as La^3^⁺, Gd^3^⁺, or Tb^3^⁺, which typically results in a shift of the emission band towards longer wavelengths [1,2,11,16,18].

Nowadays, extensive compositional engineering has effectively tuned the photoconversion properties of low- and high-entropy garnets, which has led to significant improvements in CCT and CRI parameters. However, this strategy falls short of addressing another crucial parameter, i.e., luminous efficacy (LE). This presents a significant challenge, particularly for phosphors in the form of single crystals, single-crystalline films, or transparent ceramics with a high-quality and homogenous structure [18,19,20]. The light conversion efficiency of these phosphors is detrimentally impacted by the total internal reflection (TIR). This phenomenon arises due to the high crystalline quality and homogeneous structure of the phosphors, which act as efficient waveguides. When light is generated within the phosphor, its propagation within the crystal ensues along distinct paths or modes. This results in a significant portion of the emitted light being trapped and re-directed within the phosphor due to repeated TIR events, ultimately hindering its extraction and reducing the overall light conversion efficiency [21,22,23]. Self-absorption losses caused by TIR materials can be significantly mitigated by carefully balancing the sample thickness and luminescent ion concentration. There exists an optimal thickness that maximizes the external photoluminescence quantum yield by allowing sufficient absorption of the excitation light while minimizing self-absorption of the emission. The optimal thickness depends on the luminescent ion doping level and the excitation wavelength [24,25]. Furthermore, the introduction of a reflective layer can enhance this effect by increasing the absorption of the excitation light while simultaneously reducing self-absorption losses. The reflective layer allows more efficient recycling of the unabsorbed pump light and the emission, potentially achieving higher external photoluminescence yields compared to photoluminescence efficiency without a reflective layer. Therefore, combining optimized sample thickness, luminescent ion concentration, and a reflective layer design provides an effective approach to mitigate detrimental self-absorption losses in materials [24,25,26]. Moreover, incorporating controlled scattering centers serves as an additional strategy to diminish self-absorption losses, thereby enhancing the luminous efficacy of phosphors. This approach can effectively reduce the influence of total internal reflection, which traps emitted photons within the crystal lattice and restricts their extraction. Diffusive scattering allows photons to escape from these loops of total internal reflection. Hence, the light scattering centers can enhance the collection of the light generated during photoconversion events by providing an escape pathway [21,22]. This approach was successfully implemented in eutectic Ce^3+^-doped Al_2_O_3_-Y_3_Al_5_O_12_ composite phosphors. In this system, the Al_2_O_3_ phase serves as the light scattering center (due to a more distinct reflective index than the Y_3_Al_5_O_12_ structure), leading to an enhancement in luminous efficacy [27,28,29,30]. However, eutectic formation presents several challenges that limit its widespread application in phosphors. Firstly, chemical compatibility between the components is crucial for achieving a homogeneous mixture. Generating the desired microstructure within eutectic compositions can be difficult, and achieving optimal performance is often restricted by a narrow range of possible compositions. Furthermore, effective optical transparency requires similar refractive indices in both phases, while thermal compatibility (similar thermal expansion coefficients) is necessary to avoid cracking at high temperatures [27,31,32,33].

The novel methodology, based on the atomic size mismatch and nonstoichiometric engineering, represents a significant advancement in achieving targeted sub-eutectic solidification. Unlike conventional synthesis relying on thermodynamic equilibrium, this approach induces kinetic barriers that facilitate the co-precipitation of metastable phases, effectively circumventing the limitations of equilibrium phase diagrams. Notably, this method facilitates the manipulation of multi-phase microstructures through the control of atomic radii mismatch and nonstoichiometric degree within the predominant phases, thereby enabling avenues for the design and development of novel materials with functional properties [8,9,21,34,35,36].

This study builds upon and elaborates on a novel methodology that uses engineered atomic radius mismatch to generate light scattering centers within the garnet structure. This innovative approach exhibits reduced limitations in the formation of sub-eutectic systems and possesses further extendibility to high-entropy oxide systems. Additionally, the control over the concentration of incompatible atoms enables tailoring of the secondary phase inclusions within the nano-micro scale. This level of control fosters the achievement of optimal optical transparency by mitigating potential complications arising from significant thermal expansion mismatch between immiscible phases on the microscale. Additionally, it enables the formation of light scattering centers within the garnet structure, which facilitates the process of light scattering and consequently enhances the extraction of light, enabling the formation of favorable light scattering properties. In the Tb_2_O_3_-Al_2_O_3_ oxide system, the thermodynamically most stable phase is perovskite TbAlO_3_ (TAP), which exhibits a congruent melting behavior. Congruent melting implies that TAP melts entirely at its melting temperature without undergoing any compositional changes. In contrast, garnet Tb_3_Al_5_O_12_ (TAG) possesses an incongruent melting point, indicating that upon melting, TAG decomposes into a liquid phase with a composition different from that of TAG and a solid phase of TAP, which remains the most stable phase in the system [37,38,39]. During the crystallization process, the initial precipitation of TAP enriches the remaining liquid with Al_2_O_3_, thereby hindering the subsequent formation of pure TAG crystals. This enrichment alters the Tb:Al ratio in the residual liquid, skewing it towards a higher aluminum content and deviating from the stoichiometric ratios of 3:5 or 1:1 required for TAG and TAP, respectively. As a result, the excess Al_2_O_3_ in the liquid crystallizes separately, leading to the formation of a eutectic perovskite–Al_2_O_3_ structure.

The thermodynamic instability and consequent incongruent melting of the TbAG garnet phase within the Tb_2_O_3_-Al_2_O_3_ oxide system necessitated the inclusion of Sc_2_O_3_ oxide to enhance the thermodynamic stability of the garnet phase. The incorporation of Sc_2_O_3_ facilitates the stabilization of the garnet phase and introduces a significant ionic radius mismatch between Al^3+^ and Sc^3+^, thereby promoting structural stability [40]. Raman spectroscopic investigations further confirmed the coexistence of garnet and perovskite phases across all investigated compositions. The Sc substitution profoundly influenced the garnet lattice, inducing *A_1g_* mode softening up to an Sc concentration of x = 2.0, followed by stabilization due to perovskite inclusion formation and associated strain effects.

This synergy is expected to exert a substantial driving force for the formation of nano-micro secondary phases within sub-eutectic inclusions, acting as efficient light scattering centers in the photoconversion process to enhance light extraction. Furthermore, Tb^3^⁺ serves as the matrix element, increasing the crystal field splitting energy levels of Ce^3^⁺ ions, leading to a red-shifted emission maximum [18]. Microstructural analysis revealed that tailoring the Sc^3+^ ion concentration facilitated a transition in the composition of secondary inclusions, transitioning from predominantly TbAlO_3_ and TbScO_3_ at low and high Sc^3+^ ion concentrations, respectively. Nevertheless, the crystal with the highest Sc^3+^ ion concentration also contained minor amounts of TbAlO_3_.

The incorporation of Sc atoms into the Ce^3^⁺-doped Y_3_Al_5_O_12_ crystal structure showed an effective approach for tuning the LE, CRI, and CCT of white LEDs. This tuning was achieved through the creation of light-scattering centers within the garnet structure and the modulation of energy transfer from Ce^3+^ to Tb^3+^ ions. By varying the Sc concentration, a wide range of CRI values from 68 to 84 and CCT values spanning 354–12,958 K were obtained. Notably, the Ce^3+^-doped Tb_3_Al_5−x_Sc_x_O_12_ crystal where x = 2.0 demonstrated the highest luminous efficacy of 114.6 lm/W, along with daylight-like white emission at a CCT of 4942 K. This optimal Sc^3+^ ion concentration shows a balance between enhancing light extraction through scattering and maintaining efficient energy transfer between Ce^3^⁺ and Tb^3^⁺ ions. The ability to fine-tune the emission properties of Ce^3^⁺-doped Y_3_Al_5_O_12_ through Sc incorporation offers significant potential for the development of high-performance white LEDs with tailored CRI, CCT, and luminous efficacy. This approach enables the fabrication of LEDs that can accurately reproduce the characteristics of natural daylight while achieving high luminous efficacy, making them suitable for various lighting applications, including indoor and outdoor environments.

## 2. Materials and Methods

### 2.1. Crystal Growth via Micro-Pulling down Technique

In single crystals of Tb_2.85_Ce_0.15_Al_5−x_Sc_x_O_12_, x = 0.5, 1.0, 1.5, 2.0, 3.0 were grown using the micro-pulling-down (μ-PD) technique within a radiofrequency inductive heating furnace [12,41]. An iridium crucible (Furuya Metal Co., Ltd., Tokyo, Japan) with a 3 mm inner diameter die facilitated growth under an inert 99.99% argon atmosphere. The <100> oriented Y_3_Al_5_O_12_ seed crystal was used to initiate a solidification process with a controlled growth rate of 0.05 mm/min. The <100> orientation in the context of Y_3_Al_5_O_12_ refers to a specific crystallographic direction within the cubic crystal lattice of the material. This orientation is aligned with one of the principal axes of the unit cell (x, y, or z axis). High-purity Tb_4_O_7_, Sc_2_O_3_, Al_2_O_3_, and CeO_2_ oxide powders (99.99%, Iwatami Corp., Tokyo, Japan) were used as raw materials.

### 2.2. PXRD, Theoretical Calculations of Garnet Lattice Constants, SEM-EDS, and Raman Spectroscopy

Powder X-ray diffraction (PXRD) analysis using a Bruker D8 DISCOVER (Billerica, MA, USA) diffractometer equipped with a Cu Kα X-ray source (λ = 1.54 Å, E = 8.04 keV) verified the crystalline phase purity of the grown crystals. Samples were obtained from the one-third rod length adjacent to the seed crystal by pulverizing them into fine powders using an agate mortar. Continuous powder diffraction patterns were acquired between 20° and 65° at a 0.02°/min scan rate. Theoretical estimation of lattice constants for the garnet structure was conducted based on the methodology outlined by Strocka et al. [42]. Ionic radii utilized in these calculations (noted with their respective coordination numbers) include Tb^3+^(VIII) = 1.04 Å, Sc^3+^(VI) = 0.745 Å, Al^3+^(VI) = 0.53 Å, and Al^3+^(IV) = 0.39 Å [40]. This analytical framework deliberately omitted the influence of the Ce dopant owing to its presence in trace concentrations. In the calculation, Sc was hypothesized to substitute Al exclusively in the VI coordination position within the garnet formula Tb_3_Al_5−x_Sc_x_O_12_, exploring substitutions where x = 0, 0.5, 1, 1.5, and 2, the latter representing the theoretical maximum for this coordination position within garnet crystals. Notably, this substitution trend is correlated with an increment in the lattice parameter *a_0_* as the Sc content increases.

Field emission scanning electron microscopy (FE-SEM, FEI Nova NanoSEM 230; Field Electron and Ion Company, Hillsboro, OR, USA) equipped with an energy dispersive X-ray spectrometer (SEM-EDS) EDAX Genesis XM4 (AMETEK, Inc. Cassatt Road, Berwyn, PA, USA) was used to determine the morphology and chemical composition of examined crystals. In the first step, the samples were included in the carbon resin (PolyFast Struers; Struers ApS, Pederstrupvej, Ballerup, Denmark) and then pressed by using an automatic mounting press CitoPress-1 (Struers ApS, Pederstrupvej, Ballerup, Denmark) to eliminate the charging effect during the SEM-EDS measurements. In the next step, the samples were subjected to mechanical polishing using SiC paper from 220- to 4000-grain (Struers, Ballerup, Denmark) to remove deformation layers and obtain a flat surface for examination. After that, prepared specimens were coated with a thin gold layer (using an Edwards Vacuum Auto 306 sputter coater system; Xiamen Lith Machine Limited, Nanshan Road, Xiamen, China) and placed in the microscope. SEM images and EDS elemental maps were performed by collecting secondary electron (SE) and characteristic X-ray signals, respectively. Nevertheless, due to limitations in the detection of trace elements by the EDS method (realistically, the element concentration should be greater than 0.1–0.2 wt%) and the differentiation between X-rays with similar energies, quantitative analysis for the Ce atoms was not performed. Therefore, EDS maps and EDS lines for the above-mentioned element were not presented and discussed in this paper.

Micro-Raman spectroscopy (μ-RS) facilitated the examination of structural alterations in the garnet phase induced by scandium substitution and enabled the investigation of the characteristics of secondary phase micro-inclusions. This analysis was conducted using an XploRA Plus HORIBA Scientific spectrometer (HORIBA Advanced Techno, Co., Ltd., Kyoto, Japan), which was coupled with an Olympus BX43 microscope (Olympus (Shenzhen) Industrial Ltd., Shenzhen, Guangdong Province, China). The system achieved a spectral resolution of approximately 3 cm^−1^, employing an Open Electrode CCD camera (HORIBA Advanced Techno, Co., Ltd., Kyoto, Japan) alongside a multistage Peltier air-cooling system as the detection mechanism. The measurement regime utilized dual laser sources, emitting at wavelengths of 638 nm (90 mW) and 785 nm (100 mW), and incorporated a 1200 gr/mm diffraction grating to facilitate the spectral analysis. Automated linear scans were implemented to systematically examine the phase composition across the entire sample diameter. Light attenuation was uniformly maintained at 10%, with focusing achieved via a 5× objective lens.

### 2.3. Optical, Luminescence, Photoconversion, and Thermoluminescence Properties

Optical absorption, photoluminescence excitation, and emission spectra were acquired at 300 K using a Shimadzu 3101PC spectrophotometer (Shimadzu Corporation, Kyoto, Japan) and Edinburgh Instruments FLS1000 spectrofluorometer (Edinburgh Instruments Ltd., 2 Bain Square, Kirkton Campus, UK), both equipped with a xenon lamp as the excitation source. Time-resolved photoluminescence decay kinetics measurements were facilitated by a picosecond 450 nm laser diode. Temperature-dependent analyses from 83 to 693 K were performed using a THMS Linkam (Linkam Scientific Instruments LTD., Salfords, UK) temperature stage coupled to the FLS1000 spectrofluorometer (Edinburgh Instruments Ltd., 2 Bain Square, Kirkton Campus, UK). Prompt luminescence decay curves were recorded by time-correlated single photon counting. The decay curves exhibit a single- and double-exponential nature and, consequently, are described using a multi-exponential function, expressed by the following equation [43,44]:

(1) $$I\left(t\right)=\sum_{i}I_{i}\exp\left(\frac{-t}{\tau_{i}}\right)+B,$$

where *I* represents luminescence intensity, *I_i_* denotes the intensity at 0 ns, *t* signifies the time, *τ_i_* is the decay time, and *B* stands for the background intensity. To quantify this, the mean decay time (*τ_mean_*) was calculated using the formula below [43,44]:

(2) $$\tau_{mean}=\frac{\sum_{i}I_{t}\tau_{i}^{2}}{\sum_{i}I_{i}\tau_{i}}$$

where *I_i_* represents the intensity, and *τ_i_* stands for the decay time value of the *i*-th component within the fit.

Thermoluminescence experiments were carried out using the Lexsyg Research Fully Automated TL/OSL Reader from Freiberg Instruments GmbH, Freiberg, Germany. The irradiation of materials was performed by LED (λ = 458 nm) source with 80 mW/cm^2^ of irradiation power for 60 s. Thermoluminescent glow curves were recorded through a 9235QB-type photomultiplier from ET Enterprises (Hamamatsu, Hamamatsu, Japan) in the range of 300–700 K. All experiments were controlled using LexStudio2 software.

Photoconversion characterization involves positioning the crystalline specimen on an illumination source equipped with a collimation lens to adapt the beam dimensions to the sample size. Excitation was provided by 445 nm radiation from a CNI laser diode and a 455 nm LED source, with the optical power of the blue excitation fixed at 1.0 W. Spectral acquisition was conducted using a Gigahertz BTS-256LED spectrometer (Gigahertz Optik GmbH, Munich, Germany) outfitted with an integrating sphere.

## 3. Results and Discussion

### 3.1. PXRD, SEM-EDS, and Micro-Raman Analysis of Radii Mismatch Effect on the Secondary Phases Formation and Atom Segregation

**Figure 1:**
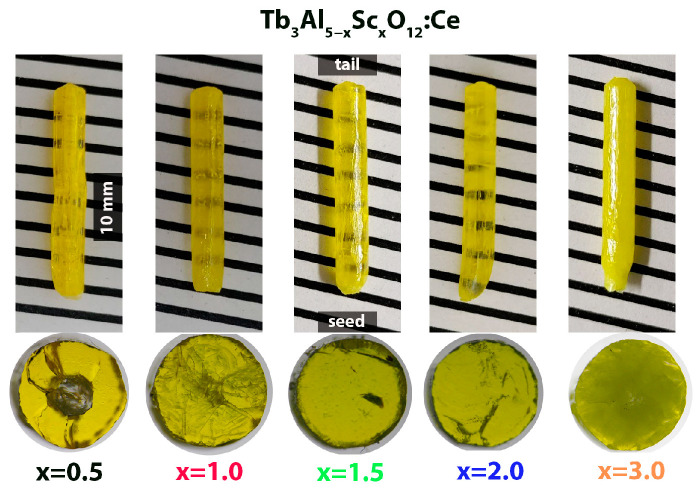
As-grown rods and polished radial plates of Tb_2.85_Ce_0.15_Al_5−x_Sc_x_O_12_ crystals with increasing Sc^3+^ ion concentration.

Figure 1 shows as-grown rods and polished radial plates of Ce^3+^-doped Tb_3_Al_5−x_Sc_x_O_12_ crystals, where x = 0.5, 1.0, 1.5, 2.0, 3.0. The presented crystals were fabricated via a controlled shaping process, yielding consistent geometric form. The maintained cylindrical shape of all crystal rods confirms the congruent melting of the material in all examined samples [9,11,34,35]. The resulting transparency demonstrated a pronounced dependence on the Sc^3+^ ion concentration.

**Figure 2:**
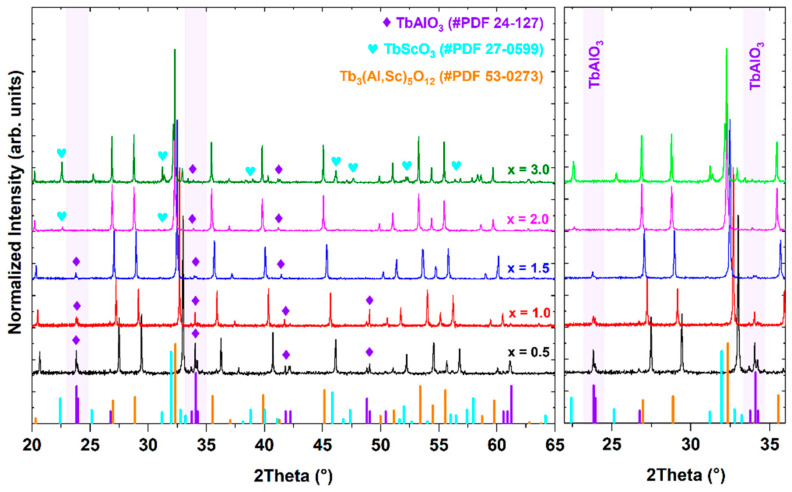
Theoretical [Tb_3_(Al,Sc)_5_O_12_—#PDF 53-0273; TbScO_3_—#PDF27-0599; TbAlO_3_—#PDF 24-127) and experimental PXRD patterns of Ce^3+^-doped Tb_3_Al_5−x_Sc_x_O_12_ crystals, where x = 0.5, 1.0, 1.5, 2.0 and 3.0.

Notably, the Ce^3+^-doped Tb_3_Al_5−x_Sc_x_O_12_ crystal, where x = 1.5, exhibits the highest degree of transparency. Conversely, crystals with Sc^3+^ ion concentrations either below or above x = 1.5 demonstrate a marked decline in transparency. This effect is most pronounced in the Ce^3+^-doped Tb_3_Al_5−x_Sc_x_O_12_ crystal (x = 3), which exhibits the lowest observed transparency. Polished radial plates were cut 8 mm away from the seed end of the crystal. The radial plates also exhibit distinct compositional heterogeneity as a function of Sc^3+^ ion concentration. As can be seen in Figure 1, the Ce^3+^-doped Tb_3_Al_5−x_Sc_x_O_12_ crystal (x = 0.5) displays a round core with contrasting coloration compared to the surrounding mantle region, which additionally contains cracks. The Ce^3+^-doped Tb_3_Al_5−x_Sc_x_O_12_ crystal (x = 1.0) also exposes numerous irregular lines that scatter the light. Notably, the Ce^3+^-doped Tb_3_Al_5−x_Sc_x_O_12_ crystals, where x = 1.5 and 2.0, exhibit the most homogenous surfaces in contrast to the Tb_2.85_Ce_0.15_Al_2_Sc_3_O_12_ crystal (x = 3.0), which possesses a cloudy morphology. These diverse patterns correlate with the formation of varying compositional domains within the examined crystals. The PXRD pattern of Ce^3+^-doped Tb_3_Al_5−x_Sc_x_O_12_ crystal (shown in Figure 2) confirms that Sc^3+^ ion concentration directly influences the formation of sub-micron-sized inclusions with distinct chemical compositions.

All examined crystals were identified as multiphasic systems with a dominant phase indexed to the garnet structure of Tb_3_(Al,Sc)_5_O_12_, confirmed by comparison to reference data (PDF No. 53-0273). The concentration and chemical composition of secondary phases varied depending on the Sc^3+^ ion concentration. Figure 2 reveals the presence of a secondary TbAlO_3_ perovskite phase (PDF No. 24-127) in all analyzed crystals. The relative intensity of the diffraction peaks assigned to the TbAlO_3_ perovskite phase systematically decreases with increasing Sc concentration. This observation suggests a corresponding decrease in the overall amount of the TbAlO_3_ perovskite phase within the samples. Notably, in Ce^3+^-doped Tb_3_Al_5−x_Sc_x_O_12_ crystals (where x ≥ 2.0), an additional TbScO_3_ perovskite phase (PDF No. 27-0599) emerges, becoming the dominant secondary phase in the Tb_2.85_Ce_0.15_Al_2_Sc_3_O_12_ crystal (x = 3.0). Table 1 depicts the non-linear variation of the lattice constant *a_0_* and unit cell volume *V_0_* with increasing Sc^3+^ ion concentration. The observed non-linear expansion of the lattice parameter, from 12.11 to 12.37 Å for Tb_2.85_Ce_0.15_Al_4.5_Sc_0.5_O_12_ (x = 0.5) and Tb_2.85_Ce_0.15_Al_2_Sc_3_O_12_ (x = 3.0) crystals, respectively, deviates from Vegard’s law (as evident from the theoretical lattice constant *a_0_*); see Table 1. This observation is consistent with the experimental PXRD patterns and confirms that the large Sc^3+^ cations have a low propensity to substitute for Al sites in octahedral coordination. Consequently, it is energetically more favorable for Sc elements to form secondary perovskite inclusions rather than incorporate them into octahedral coordination.

This phenomenon explains the observed deviation from Vegard’s law in the experimental data. Discrepancies from Vegard’s law have been well-documented, where deviations are often attributable to factors such as differences in individual component sizes and structural compatibility [45]. This preference provides strong evidence of the inherent limitations in incorporating solutes into a solid matrix when the solute atoms are significantly different in size compared to the solvent atoms [12,34]. The data suggest a compositional threshold for Sc^3+^ saturation of octahedral sites in the Ce^3+^-doped Tb_3_Al_3_Sc_2_O_12_ crystal where x = 2.0. This is evident from the significant difference between the experimental lattice constant, *a_0_* (12.20 Å), and the theoretically predicted value for full saturation (12.39 Å, Table 1). This observation implies the incomplete incorporation of Sc^3+^ cations in the Ce^3+^-doped Tb_3_Al_3_Sc_2_O_12_ crystal where x = 2.0. Conversely, in the Ce^3+^-doped Tb_3_Al_3_Sc_3_O_12_ crystal where x = 3.0, the experimental *a_0_* value (12.37 Å) approaches the theoretical value for a fully Sc^3+^-saturated lattice (12.39 Å, Table 1). This suggests a near-complete occupancy of octahedral sites by Sc^3+^ cations in Tb_3_Al_3_Sc_3_O_12_ crystal. This proximity in the lattice parameter values under conditions of excess Sc^3+^ ion concentration suggests the activation of an alternative incorporation mechanism. This mechanism facilitates near-nominal accommodation of Sc^3+^ ions within the lattice. The residual Sc atoms that exceed the substitutional capacity of the octahedral sites result in the formation of perovskite phase inclusions.

EDS elemental maps and line profile intensity analysis reveal the detailed distribution of Tb, Al, Sc, and O elements within the examined crystals. These two types of EDS techniques precisely delineate the spatial distribution and composition of secondary phase inclusions forming sub-eutectic compositions. It is worth mentioning that each of the tested samples exhibits a distinct pattern of host lattice and secondary phase distribution.

**Figure 3:**
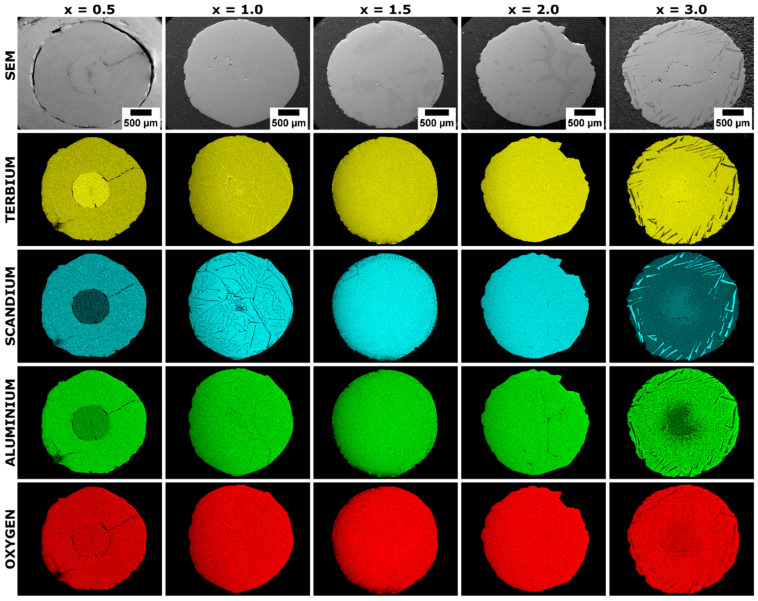
SEM images and corresponding EDS elemental distribution maps of terbium (Tb, yellow), scandium (Sc, cyan), aluminum (Al, green), and oxygen (O, red) of Ce^3+^-doped Tb_3_Al_5−x_Sc_x_O_12_ crystals, where x = 0.5, 1.0, 1.5, 2.0, and 3.0.

Figure 3 shows the multi-elemental EDS maps of the Ce^3+^-doped Tb_3_Al_5−x_Sc_x_O_12_ crystals with increasing Sc^3+^ ion concentration. In the Tb_2.85_Ce_0.15_Al_4.5_Sc_0.5_O_12_ (x = 0.5) sample, the crystal core shows enrichment with Tb, Al, and O elements, unlike the Sc element, which is depleted within the crystal core. This correlation suggests a sub-eutectic composition of mixed Tb(Al,Sc)O_3_ perovskite and Tb_3_(Al,Sc)_5_O_12_ garnet phases within the core. In contrast, the surrounding mantle exhibits a more uniform distribution of all elements, indicating its composition primarily as Tb_3_(Al,Sc)_5_O_12_ garnet. A similar phenomenon is also observed in the Tb_2.85_Ce_0.15_Al_2_Sc_3_O_12_ sample, where x is equal to 3.0. Here, the core crystal is enriched with Tb, Sc, and O elements while being depleted of the Al element. This again signifies the presence of sub-eutectic mixed Tb(Al,Sc)O_3_ perovskite and Tb_3_(Al,Sc)_5_O_12_ garnet phases. However, a key difference lies in the crystal rim, enriched with Sc atoms and depleted in Tb, Al, and O elements. This phenomenon suggests the presence of a (Tb,Sc)ScO_3_ perovskite phase within the rim inclusions. The region between the core and the rim crystal remains primarily composed of the Tb_3_(Al,Sc)_5_O_12_ garnet phase. The microstructural analysis of the Ce^3+^-doped Tb_3_Al_5−x_Sc_x_O_12_ crystal (where x = 1.0) reveals a secondary phase configuration resembling a neuronal network pattern. This sub-eutectic microstructure exhibits significant enrichment in Tb elements, with a slight depletion in Al. Importantly, Sc atoms are absent or have a very low concentration within this microstructure. These observations, along with PXRD analysis, suggest the secondary phase adopts a perovskite structure with the composition TbAlO_3_. The formation of a neuronal crystal pattern resembling a perovskite structure has also been reported in La^3+^ and Pr^3+^ co-doped Lu_3_Al_5_O_12_ crystals [12]. The incorporation of La and Pr elements, which exhibit a strong mismatch with the dodecahedral coordination of the Lu site in the garnet structure, induces significant lattice stress. This stress is released through the formation of perovskite phase inclusions within the crystal matrix. The mechanism behind this phenomenon can be attributed to the ionic size mismatch between the La^3+^ and Pr^3+^ ions and the Lu^3+^ ions in the garnet structure. The larger ionic radii of La^3+^ (1.16 Å) and Pr^3+^ (1.126 Å) compared to Lu^3+^ (0.977 Å) create local distortions and strain in the crystal lattice. To minimize the overall energy of the system, the crystal undergoes a phase separation, leading to the formation of perovskite phase inclusions that accommodate the mismatched ions. The progressive propagation of the perovskite phase inclusions from the crystal rim towards the center can be explained by the diffusion-controlled growth process. As the concentration of La^3+^ and Pr^3+^ ions increases, the driving force for phase separation becomes stronger, facilitating the growth and expansion of the perovskite inclusions. The neuronal crystal pattern observed is a result of the interconnected network of these inclusions throughout the crystal volume. The formation of the neuronal crystal pattern is highly dependent on the concentration of the mismatched La and Pr elements. It occurs only within a specific range of codopant concentrations, indicating the existence of a critical threshold for the manifestation of this unique microstructural feature. Similarly, in Ce^3+^-doped Tb_3_Al_5−x_Sc_x_O_12_ crystals, the neuronal crystal pattern emerges exclusively at a particular Sc concentration corresponding to x = 1.0. This phenomenon can be attributed to the delicate interplay between the lattice strain induced by the mismatched dopants and the thermodynamic driving force for phase separation. It should be noted that the Tb_2.85_Ce_0.15_Al_5−x_Sc_x_O_12_ crystal, where x = 1.5 and x = D 2.0, exhibit the highest degree of homogeneity. Those crystals host secondary phase inclusions that are uniformly distributed throughout their volume. In the Tb_2.85_Ce_0.15_Al_3.5_Sc_1.5_O_12_ crystal (x = 1.5), analysis reveals the enrichment of these inclusions with Tb and Al elements, as well as the observed depletion in Sc atoms. These findings, in conjunction with PXRD analysis, suggest a perovskite structure for the secondary phase with the composition TbAlO_3_. In the Tb_2.85_Ce_0.15_Al_3_Sc_2_O_12_ crystal where x = 2.0, there is an observed enrichment of Tb and Sc elements, accompanied by a reduction in Al atoms. These findings, in conjunction with PXRD analysis, suggest a perovskite structure for the secondary phase with the composition TbScO_3_.

**Figure 4:**
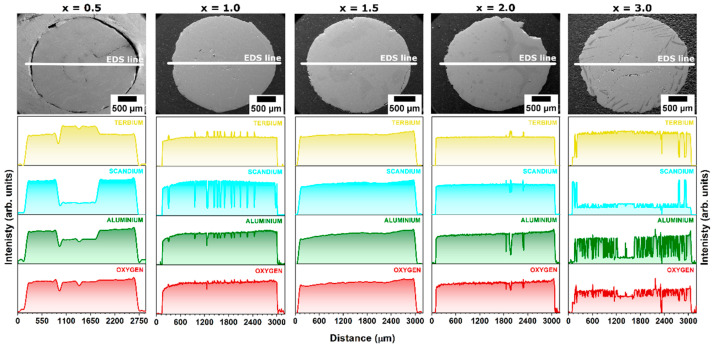
EDS line scanning profiles of terbium (Tb, yellow), scandium (Sc, cyan), aluminum (Al, green), and oxygen (O, red) recorded along a diameter of Ce^3+^-doped Tb_3_Al_5−x_Sc_x_O_12_ crystals, where x = 0.5, 1.0, 1.5, 2.0, and 3.0.

Figure 4 presents EDS line profiles of Tb, Sc, Al, and O elements for all examined crystals, which elucidates the alterations in the distribution of the above-mentioned elements resulting from the admixture of Sc elements. In the Ce^3+^-doped Tb_3_Al_5−x_Sc_x_O_12_ crystal, where x = 0.5, the crystal core demonstrates a pronounced enrichment with Tb elements, concurrently manifesting a depletion in Sc and Al atoms. Furthermore, the concentration gradient between the rim and the core of the crystal is markedly more pronounced for Sc elements in comparison to Al elements. This observation is in alignment with data procured from XRD analysis, which corroborates the predominance of the TbAlO_3_ perovskite phase as the primary inclusion. In the Ce^3+^-doped Tb_3_Al_5−x_Sc_x_O_12_ crystal where x = 1.0, there is a discernible series of amplified intensities for both Tb and Sc elements, which inversely correlates with the diminished intensities observed for Sc and Al elements. Furthermore, the variation in concentration observed for Sc elements is significantly more pronounced compared to Al atoms. This pattern offers insights into the radial distribution of secondary phase inclusions within the crystal structure and substantiates their predominant composition as TbAlO_3_. The Tb_2.85_Ce_0.15_Al_3.5_Sc_1.5_O_12_ crystal (x = 1.5) demonstrates the most uniform radial distribution of the analyzed elements among the samples evaluated. A similar phenomenon is also observed in the Tb_2.85_Ce_0.15_Al_3.5_Sc_1.5_O_12_ crystal (x = 2.0); however, randomly distributed spots with higher concentrations of Tb and Sc atoms were recorded. This observation supports the contrasting trend whereby, at higher Sc concentrations, inclusions of the TbScO_3_ perovskite phase are formed. In the Ce^3+^-doped Tb_3_Al_5−x_Sc_x_O_12_ crystal, where x = 3.0, the periphery of the crystal is markedly enriched with Sc elements and exhibits a depletion in Tb elements, alongside an almost complete absence of Al elements. This observation corroborates the presence of regions containing the TbScO_3_ perovskite phase. Additionally, substantial variations in the intensities of Sc, Al, and O elements throughout the crystal volume indicate the existence of micro-sized eutectic mixtures of garnet and perovskite phases.

**Figure 5:**
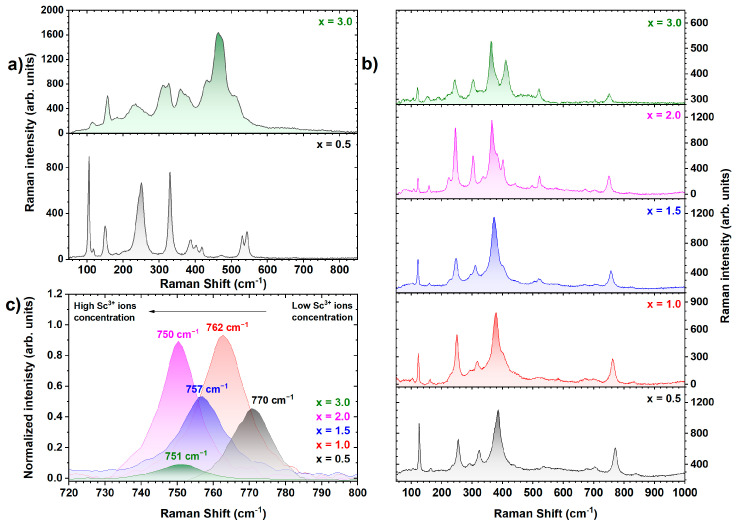
(**a**) Raman spectra acquired at an excitation wavelength of 638 nm probing the secondary phase inclusions present in the Ce^3+^-doped Tb_3_Al_5−x_Sc_x_O_12_ crystals, where x = 0.5 and 3.0 (300 K); (**b**) Raman spectra acquired at an excitation wavelength of 785 nm, probing the garnet matrix of Ce^3+^-doped Tb_3_Al_5−x_Sc_x_O_12_ crystals, where x = 0.5, 1.0, 1.5, 2.0, and 3.0; (**c**) the *A_1g_* mode peak energy position at 770 cm^−1^ for Tb_2.85_Ce_0.15_Al_4.5_Sc_0.5_O_12_ as a function of Sc^3+^ ion concentration.

Raman spectroscopic measurements confirm the coexistence of garnet and perovskite phases in all examined crystals. Corroborated by PXRD and EDS analyses, the presence of TbAlO_3_ perovskite as a predominant secondary phase inclusion is also evident from the Raman inspection (see Figure 5a, lower panel). Notably, the Raman spectrum collected on the main central inclusion of the Tb_2.85_Ce_0.15_Al_4.5_Sc_0.5_O_12_ crystal (x = 0.5) exhibits intense modes at 100, 113, 146, 247, 325, 381, 397, 414, 473, 525, and 538 cm^−1^, which are characteristic fingerprints of the orthorhombic perovskite structure (*Pnma* space group) of TbAlO_3_ [45]. In contrast, the Raman spectrum of the Ce^3+^-doped Tb_3_Al_5−x_Sc_x_O_12_ crystal, where x = 3.0 (Figure 5a, upper panel), exhibits a distinctly different spectral profile, with well-defined modes observed at approximately 113, 156, 310, 326, 360, and 380 cm^−1^, accompanied by the most intense Raman activity occurring between 400 and 600 cm^−1^. These features are highly consistent with the TbScO_3_ perovskite phase, as can be inferred from the comparison with similar systems (DyScO_3_/GeScO_3_), exhibiting ScO_6_ octahedra instead of AlO_6_ octahedra [46]. Although a comprehensive study of the Raman activity of the TbScO_3_ phase is still lacking in the literature, the similarity of the atomic masses of Dy and Ge to that of Tb elements, coupled with the PXRD and EDS results, strongly supports this conclusion. Furthermore, the observed broadening of the detected Raman modes suggests a potentially highly defective TbScO_3_ structure, likely due to strain and stress effects.

In addition to influencing the formation of secondary phase inclusions, Sc substitution markedly impacts the garnet lattice. Figure 5b illustrates the Raman spectra obtained from the Ce^3+^-doped Tb_3_Al_5−x_Sc_x_O_12_ garnet lattice as a function of Sc^3+^ ion concentration. In the Ce^3+^-doped Tb_3_Al_5−x_Sc_x_O_12_ crystal, where x = 0.5, the characteristic Raman signature of the Tb_3_Al_5_O_12_ garnet phase is recognizable, although there is a slight shift to lower energies in the modes’ frequencies compared to those reported in the literature [47]. To elucidate this observation further, the variation of the *A_1g_* mode peak energy, which occurs at 770 cm^−1^ in the Tb_2.85_Ce_0.15_Al_4.5_Sc_0.5_O_12_ crystal (x = 0.5), is analyzed as Sc^3+^ ion concentration increases, as depicted in Figure 5c. This mode, known for its sensitivity to strain and stress effects in the garnet phase, is significantly distant from the phase matrix’s activity, thereby allowing for a precise fitting procedure [48]. A notable softening of the *A_1g_* mode is observed with increasing Sc^3+^ ion concentration, transitioning from 770 cm^−1^ to 750 cm^−1^ for the Tb_2.85_Ce_0.15_Al_4.5_Sc_0.5_O_12_ and Tb_2.85_Ce_0.15_Al_3_Sc_2_O_12_ crystals, respectively. However, the position of this mode ceases to decrease for the Ce^3+^-doped Tb_3_Al_5−x_Sc_x_O_12_ crystal, where x = 3.0. Concurrently, substantial alterations are evident around 400 cm^−1^ in the spectra, as shown in Figure 5b. These changes can be attributed to the findings from PXRD and EDS analyses, which identified an oversaturation of the octahedral sites in the Tb_2.85_Ce_0.15_Al_3.5_Sc_1.5_O_12_ crystal (x = 2.0). An alternative incorporation mechanism for excess Sc atoms facilitates the formation of perovskite phase inclusions. Consequently, for the Ce^3+^-doped Tb_3_Al_5−x_Sc_x_O_12_ crystal, where x ≥ 2.0, Sc atoms cease to occupy any crystallographic sites within the garnet lattice, thereby stabilizing the energy levels associated with their vibrational modes. In contrast, the excess Sc segregates into secondary phase inclusions, potentially introducing additional strain and stress into the main garnet matrix.

### 3.2. Thermally Stimulated Luminescence in Sub-Eutectic Systems

**Figure 6:**
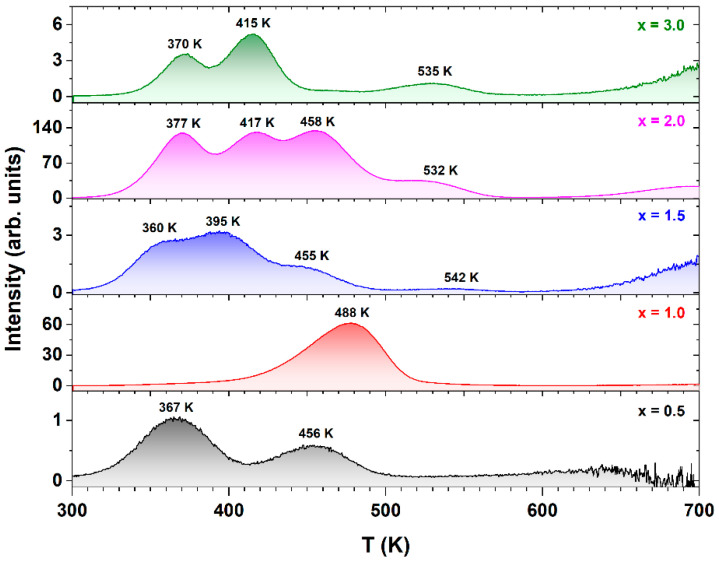
Evolution of TL glow curves in Ce^3+^-doped Tb_3_Al_5−x_Sc_x_O_12_ crystals, where x = 0.5, 1.0, 1.5, 2.0, and 3.0.

Figure 6 illustrates the thermoluminescence spectra of Ce^3+^-doped Tb_3_Al_5−x_Sc_x_O_12_ crystals irradiated for 60 s with a 458 nm wavelength at an intensity of 80 mW/cm^2^. The TL spectra demonstrate significant variations in the intensity, the number of maxima, and their positions as influenced by varying Sc^3+^ ion concentrations. The observed differences in the TL spectra are mainly due to the impact of Sc substitution within the crystal structure, which introduces defect structures and alters the crystal field environment, thus affecting the luminescent properties of the crystals [49,50,51]. For instance, the TL intensity shows noticeable variability across samples with different Sc^3+^ ion concentrations. The Ce^3+^-doped Tb_3_Al_5−x_Sc_x_O_12_ crystals, where x = 1.0 and 2.0, record the highest TL intensities, having the most effective charge carrier capture and recombination process. In contrast, Tb_2.85_Ce_0.15_Al_5−x_Sc_x_O_12_ crystals where x = 0.5, 1.5, and 3.0 manifest relatively lower intensities, likely due to less effective trapping mechanisms or saturation of the defect sites, which can quench TL. Furthermore, the number of TL peaks and their temperature positions also shift according to Sc^3+^ ion concentration. The Ce^3+^-doped Tb_3_Al_5−x_Sc_x_O_12_ crystals with higher Sc^3+^ ion concentrations where x = 1.5, 2.0, and 3.0 exhibit a more complex TL profile with a greater number of peaks at diverse temperatures, indicating the formation of varied types of trapping centers possibly stemming from increased lattice distortions and strain due to ion size mismatch and sub-eutectic microstructure formation. The variations in the TL spectra align well with the microstructural changes. As described in the structural analysis, the incorporation of Sc leads to the formation of secondary phases, such as TbAlO_3_ and TbScO_3_ perovskites, alongside the primary garnet phase. These secondary phases possess different luminescent properties and contribute to the overall TL behavior [52,53]. Additionally, the non-linear variation of the lattice constant with increasing Sc^3+^ ion concentration suggests a complex interplay between the Sc incorporation mechanism and the lattice strain. The lattice expansion and distortion influence the local crystal field environment around the luminescent centers, affecting the trapping and recombination dynamics.

The intricate microstructure and sub-eutectic character of the Ce^3+^-doped Tb_3_Al_5−x_Sc_x_O_12_ crystals pose significant challenges in accurately assigning the origin of each thermoluminescence maximum. However, by leveraging insights from prior research, it can be conclusively inferred that certain TL maxima originate from oxygen vacancies present in both the garnet and perovskite phases within the crystal structure [9,52,53,54,55]. This assertion is strongly supported by the observed TL spectra, where the position and intensity of specific maxima serve as compelling evidence for their potential origins within the complex crystal framework.

### 3.3. Absorption Spectra

**Figure 7:**
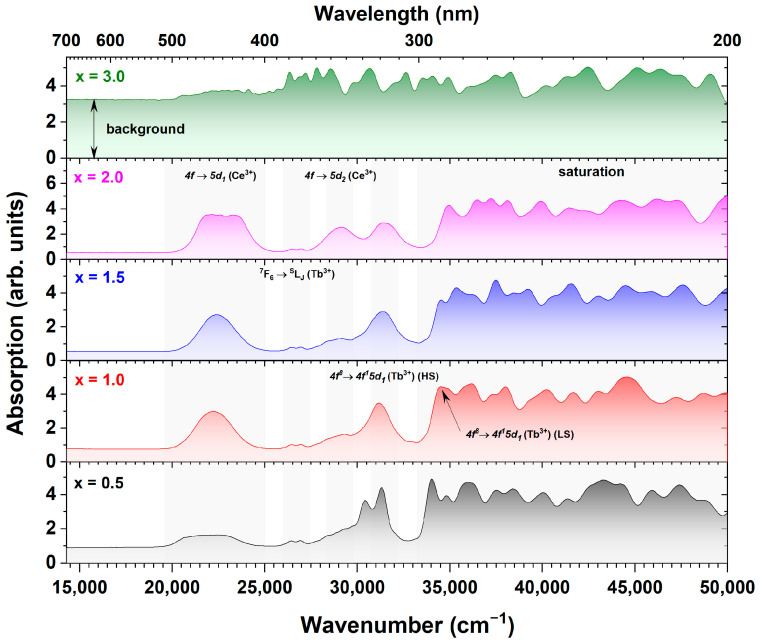
Unpolarized optical absorption spectra of Ce^3+^-doped Tb_3_Al_5−x_Sc_x_O_12_, where x = 0.5, 1.0, 1.5, 2.0, and 3.0 (300 K). The absorption spectra exhibit saturation below 300 nm due to the combined effects of the high Tb^3+^ concentration (95.5% dodecahedral site occupancy), spin-allowed *4f* → *5d* transitions, and local environmental variations within the multiphase structure. The intense background for the Ce^3+^-doped Tb_3_Al_5−x_Sc_x_O_12_ crystal where x = 3.0 arises from increased light scattering caused by reduced crystal transparency.

Figure 7 presents the unpolarized absorption spectra of Ce^3+^-doped Tb_3_Al_5−x_Sc_x_O_12_ crystals. The presented spectral features exhibit a strong dependence on the Sc^3+^ ion concentration. In the absorption spectra, Tb^3+^ ions display two primary types of transitions: spin-allowed interconfigurational *4f^8^* → *4f^7^5d* transitions and spin-forbidden intraconfigurational *4f^8^* → *4f^8^* transitions [44,56,57,58,59,60,61,62,63]. The spin-allowed *4f* → *5d* transitions, observed below 300 nm, exhibit high sensitivity to Tb^3+^ concentration, the Jahn–Teller effect, and matrix distortions [56,60,63]. The saturation observed in the absorption spectra is attributable to the high concentration of Tb^3^⁺ ions, which account for 99.5% in dodecahedral sites, along with the extended optical path lengths of 1 mm [60,63]. The high probability of spin-allowed *4f* → *5d* transitions further enhances absorption intensity, producing broad bands with high molar absorptivity and contributing to saturation [56,60,63]. The garnetperovskite eutectic character introduces local disorder around Tb^3+^ ions, causing inhomogeneous broadening in aluminum garnets with high Tb^3+^ concentrations [18].

Conversely, absorption lines around 376 nm do not show saturation due to their association with spin-forbidden *4f* → *4f* transitions, which are parity-forbidden and inherently low in intensity [56,62,64]. The *4f* electrons are shielded by the *5s* and *5p* subshells, reducing interaction with external fields and resulting in low transition probabilities [56]. This phenomenon is consistent across organic and inorganic compounds doped with Tb^3+^ ions where spin-forbidden *4f^8^* → *4f^8^* transitions are observed [11,18,56,57,58,60,64,65]. This observation is further supported by the presence of a very intense, high-spin-forbidden *4f^8^* → *4f^7^5d_1_* transition of Tb^3+^ ions centered around 320 nm. Additionally, the lines observed at approximately 376 nm can be attributed to the intraconfigurational *4f^8^* → *4f^8^* transition from the ^7^F_6_ ground state to the ^5^D_3_, ^5^L_J_, and ^5^G_J_ levels of Tb^3+^ ions [11,18,65,66]. The absorption characteristics of Ce^3+^ ions are significantly influenced by the variation in Sc^3+^ ion concentration. This influence can be attributed to the presence of a sub-eutectic microstructure, which notably perturbs the local environment surrounding the Ce^3+^ ions [11,15,18,35,36,65,67,68]. This effect is particularly pronounced in the Ce^3+^-doped Tb_3_Al_5−x_Sc_x_O_12_ crystals where x = 0.5 and 2.0, where the absorption transition at 450 nm assigned to the interconfigurational *4f* → *5d_1_* transition of Ce^3+^ ions exhibits significant broadening due to the presence of perturbed Ce^3+^ sites [18,68]. The absorption strength of the above-mentioned transition is governed by both the Ce^3+^ ion concentration and the degree of host lattice perturbation [15,67]. In the Ce^3+^-doped Tb_3_Al_5−x_Sc_x_O_12_ crystals, where x = 0.5, 1.0, and 1.5, the shape and absorption strength of the *4f* → *5d_1_* transition of Ce^3+^ ions located around 345 nm are very similar and overlap with the high-spin-forbidden *4f^8^* → *4f^7^5d_1_* transition of Tb^3+^ ions [65,67,68]. However, in the Ce^3+^-doped Tb_3_Al_5−x_Sc_x_O_12_ crystal where x = 2.0, the transition exhibits a regular shape and a significantly more intense signal. This suggests that in Ce^3+^-doped Tb_3_Al_5−x_Sc_x_O_12_ crystals, where x = 0.5, 1.0, and 1.5, a substantial portion of Ce elements incorporate into the sub-eutectic phase, leading to a reduced concentration in the dominant garnet phase. Conversely, in the Tb_2.85_Ce_0.15_Al_3_Sc_2_O_12_ crystal where x = 2.0, the Ce^3+^ ions primarily reside within the dominant garnet phase. Moreover, an elevated concentration of Sc elements leads to a decrease in the crystal field splitting energy, engendering a redshift of the *4f* → *5d_1_* transition of Ce^3+^ ions [69,70]. This, in turn, reduces the overlapping of the high-spin-forbidden *4f^8^* → *4f^7^5d_1_* transition of Tb^3^⁺ ions from the *4f* → *5d_1_* transition of Ce^3+^ ions. This reduction in overlap consequently facilitates a more distinct separation of the *4f* → *5d_1_* absorption band. These findings are consistent with the data presented in Figure 2 and Figure 3, where Ce^3+^-doped Tb_3_Al_5−x_Sc_x_O_12_ crystals where x = 0.5, 1.0, and 1.5 exhibit significantly larger quantities of sub-eutectic inclusions, while the Ce^3+^-doped Tb_3_Al_5−x_Sc_x_O_12_ crystal (x = 2.0) shows the lowest concentration of secondary phases. The Tb_2.85_Ce_0.15_Al_3.5_Sc_1.5_O_12_ crystal (x = 3.0) exhibits markedly different absorption characteristics. The absorption bands of Tb^3+^ and Ce^3+^ ions are almost indistinguishable, and the background absorption is very intense. This can be attributed to the low transparency of the sample, which significantly scatters incident radiation. The low transparency is likely caused by the presence of a high concentration of TbScO_3_ perovskite inclusions. This analysis reveals the significant role of Sc^3+^ ion concentration in tailoring the formation of secondary phases. Furthermore, it suggests that the refractive index of Tb_3_(Al,Sc)_5_O_12_ (garnet) and TbAlO_3_ (perovskite) are closer than those of Tb_3_(Al,Sc)_5_O_12_ (garnet) and TbScO_3_ (perovskite), as the former pair exhibits higher optical transparency.

### 3.4. Thermally Induced Variations in Photoluminescence Emission Spectra and Decay Kinetic

**Figure 8:**
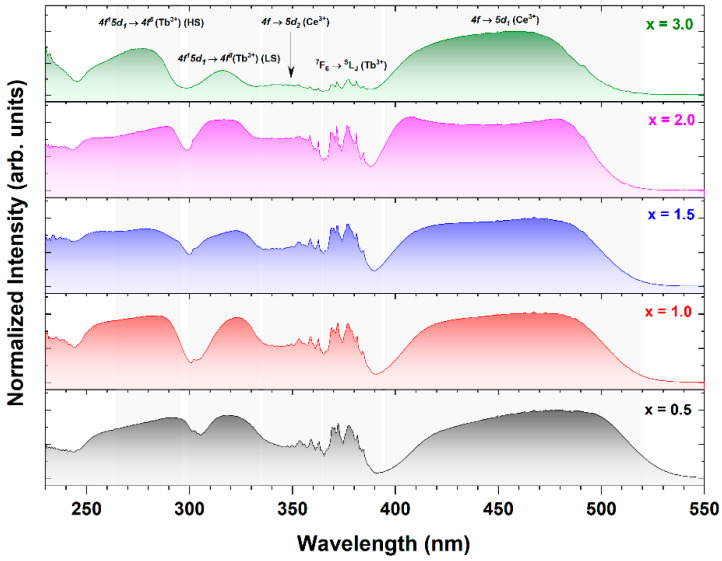
RT excitation spectra of Ce^3+^-doped Tb_3_Al_5−x_Sc_x_O_12_ crystals, where x = 0.5, 1.0, 1.5, 2.0, and 3.0 recorded at 550 nm corresponding to the *5d_1_* → *4f* transition of Ce^3+^ ions (300 K).

Figure 8 presents the excitation spectra monitored at 565 nm (attributed to the *5d_1_* → *4f* transition of Ce^3+^ ions). The identification of transitions of Tb^3+^ ions on the excitation spectra of Ce^3+^ emission signifies the presence of a Tb^3+^-to-Ce^3+^ energy transfer mechanism [18,44,69,71].

**Figure 9:**
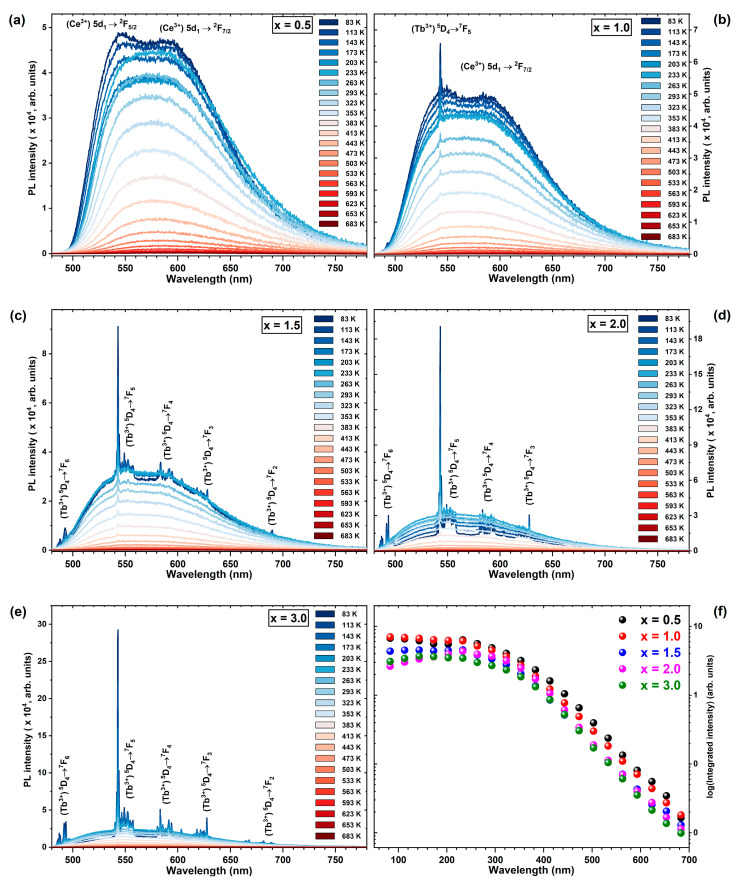
Temperature-dependent photoluminescence (**a**–**e**) emission spectra (λ_exc_ = 455 nm) and (**f**) emission spectra integrals between 480 and 750 nm of Ce^3+^-doped Tb_3_Al_5−x_Sc_x_O_12_ crystals, as measured at temperatures ranging from 83 K to 683 K, exhibiting the impact of increasing Sc^3+^ ion concentration.

The temperature dependence of the Ce^3+^ emission spectra, acquired through direct excitation in the *4f* → *5d_1_* absorption band of Ce^3+^ ions within the 83 to 683 K temperature range and in relation to increasing Sc^3+^ ion concentration, are represented in Figure 9a–e. The altered shape of the emission spectra is notably influenced by the ascending Sc^3+^ ion concentration. In the Tb_2.85_Ce_0.15_Al_4.5_Sc_0.5_O_12_ crystal (x = 0.5) across the entire temperature range, exclusive records of emission bands originating from the *5d_1_* → ^2^F_5/2,7/2_ of Ce^3+^ ions are evident. Conversely, the additional emission lines observed between 484 and 700 nm, attributed to the ^5^D_4_ → ^7^F_x_ transitions of Tb^3+^ ions, emerge on the emission spectra of Ce^3+^-doped Tb_3_Al_5−x_Sc_x_O_12_ crystals, where x ≥ 0.5 [64]. The intensity of Tb^3+^-related emission exhibits a linear rise, whereas the intensity of Ce^3+^ emission displays a linear decline with increasing Sc^3+^ ion concentration. The excitation wavelength at 455 nm does not resonate with any excitation transitions of Tb^3+^ ions, implying that the observed Tb^3+^ emission is facilitated by the Ce^3+^ ions. The Ce^3+^-to-Tb^3+^ energy transfer process is modulated by the presence of Sc atoms, as they exert an influence on this mechanism. The mechanism governing the efficiency of the Ce^3+^-to-Tb^3+^ energy transfer process is closely associated with the substantial energy shift of the *5d_1_* state of Ce^3+^ ions induced by Sc admixing. An escalating Sc^3+^ ion concentration more significantly shifts the *5d_1_* Ce^3+^ energy state to a higher energy level due to the reduction in crystal field strength, thus bringing it into closer proximity to the ^5^D_4_ energy state of Tb^3+^ ions. This minimizes the energy gap between the *5d_1_* (Ce^3+^) and ^5^D_4_ (Tb^3+^) states, facilitating efficient energy transfer from Ce^3+^ to Tb^3+^ ions. An interesting observation is that the Tb^3+^ emission lines are strongly sensitized by Ce^3+^ ions at lower temperatures, while as the temperature increases, the Ce^3+^ emission becomes more pronounced. This thermally induced redistribution of the Tb^3+^ and Ce^3+^ emission intensities can be attributed to the phonon-mediated Tb^3+^-to-Ce^3+^ back energy transfer process. This phenomenon suggests that the Ce^3+^-to-Tb^3+^ energy transfer process is more efficient than the Tb^3+^-to-Ce^3+^ energy transfer process, as the latter necessitates the assistance of host lattice phonons.

The temperature dependence of the integrated emission spectra (480–750 nm) presented in Figure 9f exhibits a minor decrease in emission intensity even for the Tb_2.85_Ce_0.15_Al_2_Sc_3_O_12_ crystal (x = 3.0). This observation is unlike other aluminum garnet crystals containing Sc elements, as Sc typically reduces the energy barrier between the *5d_1_* excited state of Ce^3+^ ions and the conduction band minimum. Consequently, the onset of quenching of Ce^3+^ luminescence typically occurs below or around 300 K [69,72,73]. This observation suggests that Ce^3+^-to-Tb^3+^ energy transfer competes with the ionization quenching mechanism. Ultimately, the Ce^3+^-to-Tb^3+^ energy transfer process overcomes the quenching mechanism. In this case, the excitation energy is not quenched through non-radiative transitions but is efficiently transferred to Tb^3+^ ions, enhancing radiative emission transitions within the Tb^3+^ ions. Hence, the loss of total excitation energy is minimal. This type of energy transfer process can be exploited to minimize luminescence quenching mechanisms in systems containing Tb and Ce elements.

**Figure 10:**
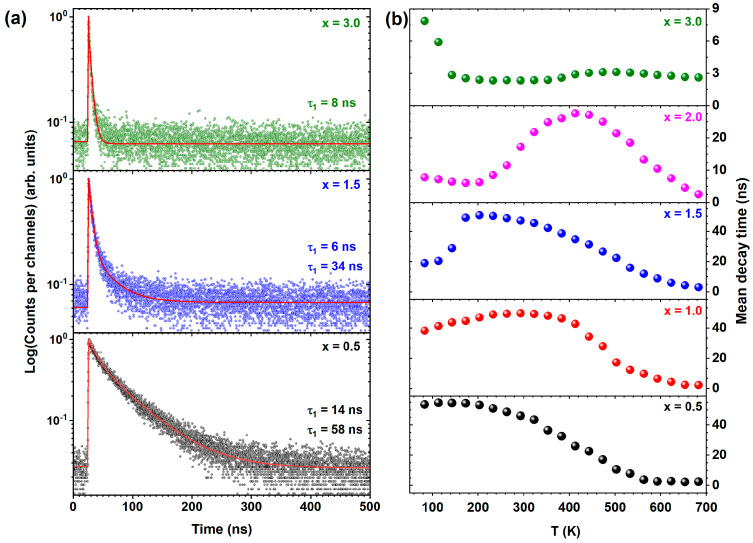
(**a**) Selected decay curves of the emission at 560 nm (λ_exc_ = 455 nm) for Ce^3+^-doped Tb_3_Al_5−x_Sc_x_O_12_ crystals, where x = 0.5, 1.5, and 3.0 recorded at 83 K; (**b**) dependence of photoluminescence decay times on temperature for emission at 560 nm (λ_exc_ = 455 nm) in Ce^3+^-doped Tb_3_Al_5−x_Sc_x_O_12_ with increasing Sc^3+^ ion concentration.

Figure 10a shows the decay times of Ce^3+^ luminescence (*λ_emi_* = 560 nm) with excitation into the *4f* → *5d_1_* transition of Ce^3+^ ions (*λ_exc_* = 455 nm) for the Ce^3+^-doped Tb_3_Al_5−x_Sc_x_O_12_ crystals where x = 0.5, 1.5, and 3.0 recorded at 83 K. The photoluminescence decay time of Ce^3+^ emission exhibits notable reduction even at a low temperature of 83 K, correlating with an increase in Sc^3+^ ion concentration. This effect is particularly marked in the Ce^3+^-doped Tb_3_Al_5−x_Sc_x_O_12_ crystal, where x = 3.0. For the Tb_2.85_Ce_0.15_Al_4.5_Sc_0.5_O_12_ crystal (x = 0.5), the decay is modeled by a double-exponential function, yielding decay values of τ_1_ = 14 ns and τ_2_ = 58 ns. Conversely, the Tb_2.85_Ce_0.15_Al_2_Sc_3_O_12_ crystal (x = 3.0) exhibits a single-exponential decay profile with a significantly reduced decay value of τ_1_ = 8 ns, in contrast to the typical decay time of 50–60 ns observed for isolated Ce^3+^ ions in garnets [3,18]. This pronounced acceleration of the first decay component further substantiates the hypothesis of energy transfer from Ce^3+^ to Tb^3+^ ions, demonstrating enhanced interaction dynamics within the host lattice.

Figure 10b shows temperature-dependent mean decay times of Ce^3+^ luminescence at 560 nm, with direct excitation at 455 nm corresponding to the *4f* → *5d_1_* transition of Ce^3+^ ions. The mean decay times of Ce^3+^ luminescence display marked variations with increasing Sc^3+^ ion concentration, closely correlating with the temperature dependence observed in the photoluminescence spectra (refer to Figure 9). These non-exponential decay characteristics of the Ce^3+^ emission, along with a pronounced acceleration of the mean decay time values with rising Sc concentration, verify the strong coupling between the *5d_1_* electronic states of Ce^3+^ and the ^5^D_4_ states of Tb^3+^, mediated through the Sc^3+^ ion concentration and the phononic environment of the host lattice. Notably, the mean decay time values accelerate from 55 ns to 8 ns in the Tb_2.85_Ce_0.15_Al_4.5_Sc_0.5_O_12_ (x = 0.5) and Tb_2.85_Ce_0.15_Al_2_Sc_3_O_12_ (x = 3.0) crystals, respectively. This substantial acceleration underscores an efficient energy transfer from Ce^3+^ to Tb^3+^ ions, a phenomenon that parallels observations in the photoluminescence spectra shown in Figure 9. With increasing Sc^3+^ ion concentration, the rate of the Ce^3+^-to-Tb^3+^ energy transfer process increases due to enhanced interaction between the *5d_1_* (Ce^3+^) and ^5^D_4_ (Tb^3+^) energy states. Moreover, in the Ce^3+^-doped Tb_3_Al_5−x_Sc_x_O_12_ crystal, where x ≥ 1.0, the dynamics of the Ce^3+^-to-Tb^3+^ energy transfer process are influenced by phonons. Specifically, at lower temperatures, the back-and-forth energy transfer between Ce^3+^ and Tb^3+^ is slightly impeded by minor energy mismatches between the *5d_1_* (Ce^3+^) and the ^5^D_4_ (Tb^3+^) energy states. Nonetheless, as the temperature elevates, phonons assist in overcoming these energy mismatches, leading to an increased rate of energy transfer between neighboring Ce^3+^ and Tb^3+^ ions. This mechanism facilitates a repetitive bidirectional Ce^3+^ ↔ Tb^3+^ energy transfer, elucidating the observed prolongation of the mean decay time at higher temperatures. This observation is in agreement with previous reports that have detailed this type of energy transfer mechanism extensively [18,44,71,74].

### 3.5. Photoconversion Characteristics

**Figure 11:**
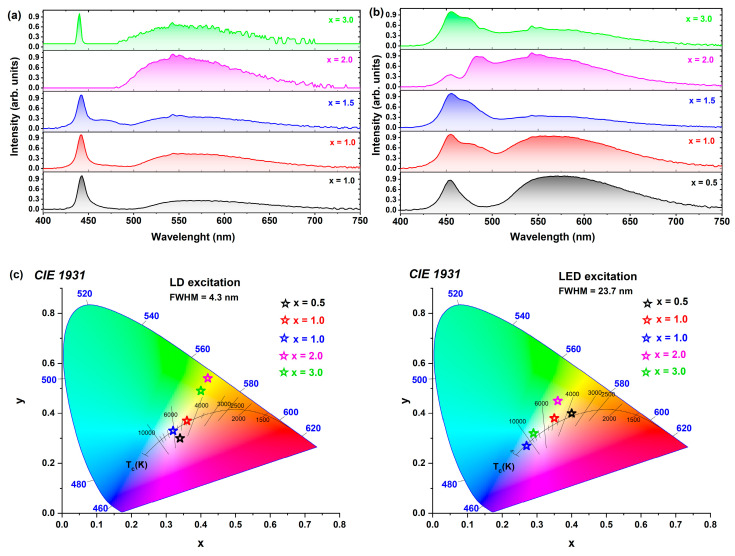
The photoconversion spectra of Ce^3+^-doped Tb_3_Al_5−x_Sc_x_O_12_ crystals delineate the impact of varying Sc^3+^ ion concentrations under (**a**) 445 nm laser diode and (**b**) 455 nm light-emitting diode excitations; (**c**) representation of observed emission spectra for LED and LD plotted on the CIE 1931 chromaticity diagram.

Figure 11a–c present a comprehensive investigation into the photoconversion properties of Ce^3+^-doped Tb_3_Al_5−x_Sc_x_O_12_ crystals, using both a laser diode and a light-emitting diode as excitation sources. The selection of these sources was based on the absorption band characteristics of Ce^3^⁺ ions, which exhibit a maximum at approximately 455 nm. Notably, the width and shape of this absorption band are significantly influenced by the concentration of scandium elements incorporated into the crystal lattice. To ensure optimal excitation, the spot size was carefully adjusted to match the diameter of the crystals.

Several key factors were found to have a profound impact on the photoconversion properties of Ce^3+^-doped Tb_3_Al_5−x_Sc_x_O_12_ crystals. In addition to the position of the absorption band maximum, the inhomogeneity of the crystals and the intrinsic scattering of the excitation radiation, which is dependent on the scandium concentration, play crucial roles. Furthermore, the energy transfer between Ce^3+^ and Tb^3+^ ions results in an additional emission band in the green spectral range, further contributing to the overall photoconversion efficiency. The LED excitation source demonstrated enhanced photoconversion parameters compared to the LD, attributable to its better alignment with the absorption band maximum and broader excitation band half-width. Across all crystals, the luminescence efficiency (lm/W) is consistently higher when using the LED source. The Ce^3+^-doped Tb_3_Al_5−x_Sc_x_O_12_ crystal, where x = 2.0, exhibits the highest efficiency equal to 114.5 lm/W, which can be ascribed to the presence of light scattering centers, enhanced internal reflection, and the broadening of the absorption band [8,12] These factors collectively lead to more efficient absorption of the excitation radiation and, consequently, more efficient emission from both Ce^3^⁺ and Tb^3^⁺ ions. In contrast, Ce^3+^-doped Tb_3_Al_5−x_Sc_x_O_12_ where x = 3.0, due to its lowest transparency, displayed the lowest efficiency of 30 lm/W under LED excitation. The incorporation of Sc^3+^ ions into Ce^3+^-doped Tb_3_Al_5−x_Sc_x_O_12_ crystals enables the effective modulation of the CRI and CCT values. This control is achieved through the intentional introduction of excitation scattering centers and the ability to adjust the ratio between the excitation source intensity and phosphor emission. The highest CRI values were obtained for the Tb_2.85_Ce_0.15_Al_3.5_Sc_1.5_O_12_ (x = 1.5) and Tb_2.85_Ce_0.15_Al_2_Sc_3_O_12_ (x = 3.0) crystals, reaching 83.6 and 84.3, respectively. Moreover, by varying the Sc^3+^ ion concentration, the color temperature of the emission could be tuned over a wide range, from 354 to 12,958 K; see Figure 11c and Table 2. Notably, the Ce^3+^-doped Tb_3_Al_5−x_Sc_x_O_12_ crystal where x = 2.0 exhibits the highest luminous efficiency (114.5 lm/W), emitting light with a CCT of 4942 K, corresponding to daylight white [75]; see Figure 11c and Table 2.

Table 2 summarizes the CCT, CRI, and LE parameters for all Ce^3+^-doped Tb_3_Al_5−x_Sc_x_O_12_ crystals.

**Figure 12:**
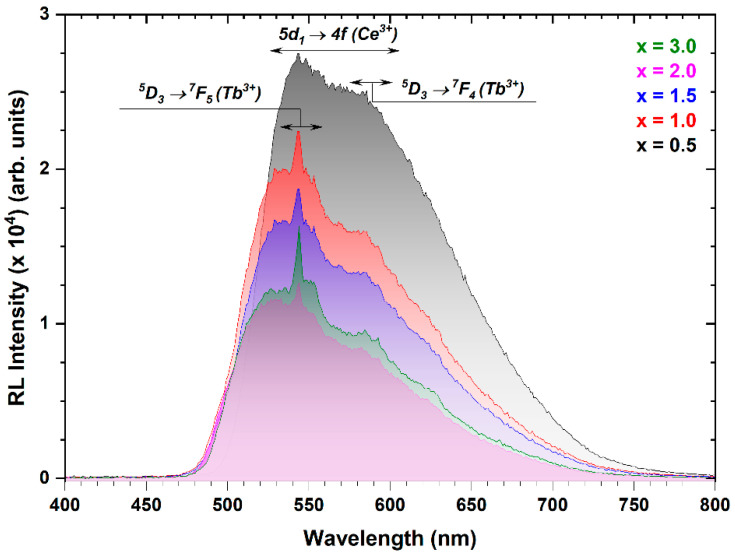
Radioluminescence spectra of Ce^3+^-doped Tb_3_Al_5−x_Sc_x_O_12_ crystals, where x = 0.5, 1.0, 1.5, 2.0, and 3.0.

Figure 12 presents a comparison of room-temperature radioluminescence spectra for Ce^3+^-doped Tb_3_Al_5−x_Sc_x_O_12_ crystals with varying Sc^3+^ ion concentrations recorded at 300 K. Notably, the RL spectra lack luminescence in the UV spectral range typically associated with diverse antisite defects and perovskite-phase-related luminescence, commonly observed in systems containing perovskite phase inclusions [35,36,68,70,76,77]. This absence of both defect-induced emission and Ce^3+^ luminescence within the perovskite phase inclusions can be ascribed to two primary factors: (1) the relatively high emission intensity of Ce^3^⁺ ions in the garnet phase and (2) the highly efficient energy transfer between Tb^3+^ and Ce^3+^ ions. Consequently, the intense Ce^3+^ emission from the garnet phase effectively masks any potential luminescence arising from other centers within the crystal.

The emission intensity of Ce^3^⁺ ions from the *5d_1_* energy state (centered at 550 nm) progressively decreases with increasing Sc^3+^ ion concentration, while the opposite trend is observed for the ^5^D_4_ → ^7^F_5_ (544 nm) and ^5^D_4_ → ^7^F_5_ (584 nm) transitions of Tb^3+^ ions. This discrepancy arises from Sc-induced changes in the position of the *5d_1_* energy state of Ce^3+^ ions, disrupting the resonance efficiency for energy transfer between them. Specifically, Sc^3+^ cations cause a blue shift of the *5d_1_* energy state of Ce^3+^ ions, detuning it from the optimal resonance with the ^5^D_3_ level of Tb^3+^ ions. This hinders the transfer of excitation energy from Tb^3+^-to-Ce^3+^, leading to a decrease in Ce^3+^ emission intensity. Consequently, the remaining excitation energy is primarily emitted by Tb^3+^ ions, resulting in stronger Tb^3+^ emission observed with increasing Sc^3+^ ion concentration. These data are consistent with photoluminescence spectra.

## 4. Conclusions

This research presented a novel methodology for strategically engineering the luminescence and photoconversion properties of Ce^3+^-doped Tb_3_Al_5−x_Sc_x_O_12_ crystals through atomic size mismatch engineering. The innovative approach exploited the incorporation of Sc atoms to induce the formation of sub-eutectic inclusions within the garnet structure, acting as efficient light scattering centers. Comprehensive microstructural analyses, including powder X-ray diffraction (PXRD), scanning electron microscopy combined with energy-dispersive X-ray spectroscopy (SEM-EDS), unveiled the intricate interplay between Sc^3+^ ion concentration and the evolution of secondary perovskite phases. Microstructural analyses unveiled a compositional threshold contingent upon the Sc^3+^ ion concentration, wherein for the Ce^3+^-doped Tb_3_Al_5−x_Sc_x_O_12_ crystals where x ≤ 1.5, the TbAlO_3_ perovskite phase manifested as the predominant secondary phase coexisting with the primary Tb_3_(Al,Sc)_5_O_12_ garnet phase. Conversely, for the Ce^3+^-doped Tb_3_Al_5−x_Sc_x_O_12_ crystals where x ≥ 2.0, the TbScO_3_ perovskite phase emerged as the dominant secondary inclusion phase within the predominant Tb_3_(Al,Sc)_5_O_12_ garnet matrix. The Raman spectroscopy investigation further confirmed the coexistence of garnet and perovskite phases, with the specific perovskite type dependent on the Sc^3+^ ion concentration. Lower concentrations (x = 0.5) favored the formation of TbAlO_3_, while higher concentrations (x ≥ 2.0) resulted in the formation of TbScO_3_. The substitution of Sc significantly impacted the garnet lattice, inducing a softening of the *A_1g_* Raman mode at lower concentrations due to strain effects. However, at higher Sc^3+^ ion concentrations, saturation within the garnet lattice led to the formation of TbScO_3_ perovskite inclusions. This effectively stabilized the vibrational modes of the remaining garnet structure. The non-linear variation of the j lattice constant with increasing Sc^3+^ ions deviated from Vegard’s law, indicating a low propensity for Sc substitution into the octahedral sites of the garnet structure. Instead, Sc atoms preferentially formed secondary perovskite inclusions, highlighting the complex interplay between solute incorporation mechanisms and lattice strain. The incorporation of Sc atoms facilitated the modulation of energy transfer dynamics between Ce^3+^ and Tb^3+^ ions, enabling the enhancement of luminescence and mitigation of quenching mechanisms. Photoluminescence analyses revealed that increasing Sc^3+^ ion concentration induced a substantial energy shift in the *5d_1_* state of Ce^3+^ ions, bringing it into resonance with the ^5^D_4_ level of Tb^3+^ ions and promoting efficient energy transfer from Ce^3+^ to Tb^3+^. This energy transfer process effectively competed with and overcame the ionization quenching mechanism, minimizing the loss of excitation energy and maintaining the overall luminescence efficiency. Notably, the energy transfer dynamics were found to be temperature-dependent, governed by phonon-mediated back-and-forth energy transfer between Ce^3+^ and Tb^3+^. At lower temperatures, the energy transfer from Ce^3+^ to Tb^3+^ was more efficient, while at higher temperatures, the back-transfer from Tb^3+^ to Ce^3+^ became more pronounced, facilitated by host lattice phonons. The Sc admixing enabled precise control over the color rendering index (CRI) and correlated color temperature (CCT) by introducing light scattering centers and manipulating the Ce^3+^-to-Tb^3+^ energy transfer rate. Optimal CRI values of 83.6 and 84.3 were observed for the Tb_2.85_Ce_0.15_Al_3.5_Sc_1.5_O_12_ (x = 1.5) and Tb_2.85_Ce_0.15_Al_2_Sc_3_O_12_ (x = 3.0) crystals, respectively, demonstrating the material’s ability to maintain high color fidelity. Furthermore, adjusting the scandium Sc^3+^ ion concentration allowed for extensive tuning of the emission color temperature from 3545 K to 12,958 K, showing significant flexibility in light characteristic manipulation. Notably, the Ce^3+^-doped Tb_3_Al_5−x_Sc_x_O_12_ crystal where x = 2.0 achieved the highest luminous efficacy of 114.5 lm/W under LED excitation with desirable daylight white emission (CCT equal to 4942 K), making it suitable for energy-efficient lighting applications requiring natural color perception. The versatility of the atomic size mismatch engineering approach extended beyond the explored Ce^3+^-doped Tb_3_(Al,Sc)_5_O_12_ system, offering a robust platform for the design and development of novel functional materials with tailored optical properties. By leveraging the principles of controlled solute incorporation and induced sub-eutectic microstructures, it could unlock new frontiers in material science, fostering the creation of advanced materials with unprecedented functionalities and applications across diverse fields, including optoelectronics, solid-state lighting, biomedical imaging, and beyond.

## Figures and Tables

**Table 1:** Comparison of theoretical and experimental host lattice constants (*a_0_*) and unit cell volume (*V_0_*) values for Ce^3+^-doped Tb_3_Al_5−x_Sc_x_O_12_ crystals across varying Sc concentrations (x = 0.5, 1.0, 1.5, 2.0, 3.0).

Sc^3+^ Ions Concentration	Theoretical Lattice Parameters		Experimental Lattice Parameters		*a*_theo_–*a*_exp_	*V*_theo_–*V*_exp_
	a (Å)	V (Å^3^)	a (Å)	V (Å^3^)		
0.5	12.13	1788.35	12.11	1775.95	0.03	12.39
1.0	12.22	1826.18	12.22	1828.22	−0.01	−2.04
1.5	12.30	1864.54	12.21	1820.65	0.10	43.88
2.0	12.39	1903.43	12.20	1817.87	0.19	85.56
3.0	n/a ^1^	n/a ^1^	12.38	1897.00	n/a ^1^	n/a ^1^

^1^ The theoretical host lattice constants and unit cell volume values for Ce^3+^-doped Tb_3_Al_2_Sc_3_O_12_ crystal were not calculated due to the intrinsic structural limitations of the garnet lattice, which provides only two available octahedral coordination sites to accommodate the Sc element.

**Table 2:** The calculated CRI, CCT, LE, and CIE 1931 parameters for the photoconversion spectra of Ce^3+^-doped Tb_3_Al_5−x_Sc_x_O_12_crystals, where x = 0.5, 1.0, 1.5, 2.0, and 3.0.

Parameters	Sc^3+^ Ion Concentration in the Ce^3+^-Doped Tb_3_Asl_5−x_Sc_x_O_12_ Crystals									
	0.5		1.0		1.5		2.0		3.0	
	LD	LED	LD	LED	LD	LED	LD	LED	LD	LED
CRI	69.9	69.0	68.5	79.3	77.6	83.6	-	65.6	60.9	84.3
CCT (K)	4684	3545	4411	4742	5922	12,958	-	4942	4161	7635
LE (lm/W)	60.0	87.4	94.7	99.7	70.7	74.3	48.4	114.6	17.6	30.3

## Data Availability

The original contributions presented in the study are included in the article, further inquiries can be directed to the corresponding author.

## Abbreviations

CCD	Charge-Coupled Device
CRI	Color Rendering Index
LE	Luminous Efficacy
mW	Milliwatt
PDF	Powder Diffraction File
μ-PD	Micro-Pulling-Down
SE	Secondary Electron
TAP	Terbium Aluminum Perovskite (TbAlO_3_)
TIR	Total Internal Reflection
OSL	Optically Stimulated Luminescence
YAG	Yttrium Aluminum Garnet (Y_3_Al_5_O_12_)
CCT	Correlated Color Temperature
LED	Light-Emitting Diode
LD	Laser Diode
nm	Nanometer
PXRD	Powder X-ray Diffraction
RL	Radioluminescence
SEM-EDS	Scanning Electron Microscopy equipped with an Energy Dispersive X-ray Spectrometer
TAG	Terbium Aluminum Garnet (Tb_3_Al_5_O_12_)
TL	Thermoluminescence
UV	Ultraviolet
μ-RS	Micro-Raman Spectroscopy
